# Occupational stress and associated factors among clinical nurses caring for COVID-19 patients in a Vietnamese tertiary hospital

**DOI:** 10.1371/journal.pone.0309028

**Published:** 2024-08-15

**Authors:** Phu Dinh Vu, Thuong Thi Nguyen, Duyet Van Le

**Affiliations:** 1 Intensive Care Unit, National Hospital for Tropical Diseases, Hanoi, Vietnam; 2 Microbiology–Molecular Biology Department, National Hospital for Tropical Diseases, Hanoi, Vietnam; Kwame Nkrumah University of Science and Technology, GHANA

## Abstract

**Background:**

Nursing professional is one of the most stressful jobs, particularly during the COVID-19 pandemic. When caring for COVID-19 patients, nurses face challenging conditions and limited resources, as well as the fear of infecting themselves and their families, putting them at risk for depression, anxiety, and insomnia. The purpose of this study was to determine the frequency, sources, and risk factors for occupational stress among clinical nurses caring for COVID-19 patients in a Vietnamese tertiary hospital.

**Methods:**

A cross-sectional survey was conducted among all clinical nurses (184 nurses) at a tertiary hospital in Vietnam from March 15 to April 15, 2021. A questionnaire was used for collecting data. Data analysis was done by descriptive statistics, bivariate and multivariate logistic regressions. Risk factors were identified by adjusted odds ratio with 95% confidence interval and P values less than 0.05.

**Results:**

The survey was completed by 89.7% (165/184) of clinical nurses. Most participants were female (85.5%) and ≤ 40 years old (97.6%). Overall, participants reported a medium stress level with an Extended Nursing Stress Scale (ENSS) mean score of 1.79 points, and 32.1% had occupational stress. Prevalence of occupational stress among participants caring for COVID-19 patients (34.0%) was not statistically significant difference with that among those who did not (29.4%). Nurses’ occupational stress in emergency and intensive care units (50.0%) was substantially higher than that in the other departments (11.7%). The most stressors for participants were difficulties connected to inadequate emotional preparedness, patients and families, and death and dying, with subscale mean scores of 1.97, 1.88, and 1.88 points, respectively. In multivariate analysis, working at an emergency and intensive care unit (OR 4.97), usually or more frequently feeling heavy duty for patients (OR 3.17), and income decrease (OR 3.03) were risk factors associated with occupational stress.

**Conclusion:**

One-third of clinical nurses at a tertiary hospital experienced occupational stress, with highest rate occurred at emergency and intensive care units. Nurses’ working conditions at emergency and intensive care units should be essentially addressed to improve nurses’ occupational stress.

## Introduction

Occupational nursing stress is a negative physical and emotional response that occurs when a nurse’s capabilities, resources, and needs are incompatible with their job requirements [1]. Occupational stress among nurses is now fairly frequent, ranging from 10% to 70% [2].

COVID-19 emerged from the Wuhan, China, in December 2019, then has rapidly become the global pandemic with 772,138,818 confirmed cases and 6,985,964 deaths by 6^th^ December 2023 [3]. The appearance of COVID-19 had a significant impact on the healthcare sector and healthcare employees, particularly nurses. They had to wear complete Personal Protective Equipment for an extended amount of time each time [4, 5], as well as be ostracized and discriminated against by the population [6], due to the quantity of COVID-19 patients overwhelming any healthcare system and the capability of all healthcare staff where the pandemic happened. Otherwise, nurses faced a high risk of COVID-19 infection while providing direct patient care, endangering their health [7]. The fear of the COVID-19 infection may enhance the work stress of nurses [8, 9]. All these overload factors could exacerbate nurses’ occupational stress [6, 10, 11].

Prior to the COVID-19 outbreak in Vietnam, several studies employing the depression anxiety stress scale– 21 (DASS-21) found that the prevalence of occupational stress among nurses ranged from 19.6% to 35.1% [12–14]. Other studies utilizing the Extended Nursing Stress Scale (ENSS) reported that nurses experienced low to moderate occupational stress, with an ENSS mean score ranging from 1.76 to 2.14 points [15–17].

The COVID-19 epidemic began in Vietnam in early 2020 and reached numerous peaks in various areas until May 2021, when it spread constantly from several provinces in the north to Ho Chi Minh City and nearly all provinces in the south. Following that, the pandemic spread rapidly throughout the country. More than 11.619.000 people have been detected with COVID-19, with over 43.200 people dying in Vietnam [18].

Several studies on stress among frontline healthcare workers in the first (in early 2020) and second waves of COVID-19 in Vietnam found that stress levels ranged from 7.7% to 44.6% [19–21]. All these studies used Depression Anxiety Stress Scale ‐ 21 to determine the common mental health impact of COVID-19 on all frontline healthcare workers including nurses. This scale was not suitable for assessing occupational stress risk in nurses. The Expanded Nursing Stress Scale (ENSS) has been used in many studies to measure stress factors related to the work of nurses because it is a specialized scale to measure the stress of nurses [6].

NHTD is the largest frontline hospital in Vietnam that admits and treats COVID-19 patients. To date, there have been no studies evaluating the stress level of clinical nurses directly caring for COVID-19 patients at NHTD; therefore, it is necessary to evaluate the current situation, sources, and risks. The present study aimed to investigate the prevalence, sources, and risk factors of occupational stress among clinical nurses directly caring for COVID-19 patients at NHTD to provide intervention solutions or support to reduce stress.

## Methods

### Study design and setting

A cross-sectional study was conducted at the National Hospital for Tropical Diseases from March 15 to April 15, 2021. The hospital is located in Hanoi Capital, with two separate institutions: one at 78 Giai Phong Street, Dong Da District, and one at Thon Bau, Kim Chung Community, Dong Anh District. During the pandemic, the facility at Thon Bau was solely utilized for COVID-19 patients, while the other was used for patients with other infectious diseases as usual. During the study period, there were total 260 nurses working at the National Hospital for Tropical Diseases.

### Study population

All clinical nurses, who were fulltime employees and gave directly care of inpatients at clinical departments of National Hospital for Tropical Diseases during study period, were the source population.

### Inclusion and exclusion criteria

All clinical nurses at NHTD who met all three of the following criteria were included in the study: (1) having a period of 12 months or more working at NHTD; (2) Giving directly inpatient care at clinical departments; and (3) agreement to sign a consent form.

Nurses who fulfilled above criteria but could not contact directly to interview in the study period were excluded.

### Sample and sampling technique

The sample size was calculated using mean proportion of nurses’ occupational stress from available studies in Vietnam (27.3%) [12–14]. As a result, nurses’ occupational stress prevalence of 27.3% with 95% confidence interval of 5.0%, the sample size needed at least 156 nurses to meet the given precision. There was a total of 184 clinical nurses directly providing care for COVID-19 patients at NHTD; however, only 165 nurses agreed to participate in the study (signing the consent form and completing the interview). Therefore, 165 qualified clinical nurses were selected to participated in this study.

### Data collection instrument

A questionnaire was used as the research tool, which included questions on general information, the Extended Nursing Stress Scale (ENSS), and working conditions and income (more details are presented in S2 File). To assess nurses’ occupational stress, the Extended Nursing Stress Scale, developed by Susan E. French et al. from the Nursing Stress Scale developed by Pamela Gray‑Toft and James Anderson, was utilized [22]. The scale consists of 57 elements divided into nine subscales. These are: (1) Death and dying (7 items); (2) Conflict with physician (5 items); (3) Inadequate emotional preparation (3 items); (4) Problems with peer support (6 items); (5) Problems with supervisors (7 items); (6) Workload (9 items); (7) Uncertainty concerning treatment (9 items). (8) Patients and their families (8 items); (9) Discrimination (3 items). Each item was rated on a 5‑point Likert scale, with 1 being “never stressful”, 2 being “sometime stressful”, 3 being “usually stressful”, 4 being “always stressful” and 0 being “does not apply”. With Cronbach’s alpha (α), the overall ENSS dependability was 0.96. Individual subscale reliability ranged from α = 0.88 (supervisors difficulties) to α = 0.65 (discrimination) [22]. In this study, the reliability of the Vietnamese ENSS version was assessed by a pilot of 30 participants and reassessed when the study finished; Cronbach’s alpha (α) for ENSS was 0.97, and the subscales varied from 0.60 with Discrimination to 0.89 with Problems with supervisors (Table 1).

**Table 1:** Reliability test of the extended nursing stress scale.

Stressors	Number of items	Scale mean	Scale variance	Cronbach’s alpha
***Extended nursing stress scale***	57	101.89	729.00	0.975
***Subscales***				
(1) Death and dying	7	13.18	12.67	0.812
(2) Conflict with Physicians	5	9.06	6.83	0.763
(3) Inadequate emotional preparation	3	5.65	2.53	0.596
(4) Problems with peer support	6	9.53	8.88	0.848
(5) Problems with supervisors	7	11.34	15.14	0.894
(6) Workload	9	16.34	21.54	0.879
(7) Uncertainty concerning treatment	9	16.85	25.38	0.889
(8) Patients and families	8	15.73	20.03	0.862
(9) Discrimination	3	4.21	3.57	0.826

The ENSS core of a participant (range 0–228) was calculated by summing the scores from all items. There are no particular cutoff scores for determining whether or not someone has occupational stress. Higher stress levels were perceived as a result of high scores. In this study, the mean score (for each item, subscale, or total ENSS) was used to identify stressful level which participants perceived from that item, subscale, or total ENSS, as follows: Mean score = 1.00 indicates no stress for participants; mean score >1.00 and <2.00 indicates a low stressful level; mean score ≥ 2.00 and < 3.00 indicates a moderate stressful level; and mean score > 3.00 indicates a high stressful level. Mean score of total ENSS (57 items) was used to classify occupational stress of participants. Participants with total ENSS mean score of ≥ 2.00 points were recognized as having occupational stress and participants with total ENSS mean score less than 2.00 points were classified as not having occupational stress [1].

### Data analysis

The study data were entered into SPSS software (version 26). Descriptive statistics was used to calculate proportion and frequency for categorical variables; mean and standard deviation for quantitative variables. Logistic regression was used to explore association between independent variables and nurses’ occupational stress. The dependent variable of occupational stress in nurses was determined with an average score of ≥ 2.00 points. Independent variables included in the analysis included age group (categorized < 30, ≥ 30 years); gender (male, female); Marital status (married, living alone or divorced); take care of children < 5 years old (yes, no); have a chronic diseases (yes, no); Educational level (bachelor, associate); Work experience (≤ 5 years, 5–10 years, and >10 years), working department (emergency department or intensive care unit, the other departments); number of days on duty per month (none, <7 days, and ≥ 7 days); direct care of covid 19 patients (yes, no); worry about covid 19 (yes, no); encouragement (sometime or less, usually or more); feeling heavy duty for patients (usually or more, sometime or less); and job satisfaction (not yet, yes); decreased income (yes or no). Multivariable logistic regression was performed simultaneously with independent variables to control for possible confounding factors. Crude and adjusted ORs were calculated and compared with 95% confidence intervals to determine each association between independent and dependent variables. Adjusted odds ratio (OR) is statistically significant when the confidence interval does not contain the value 1.

### Ethical considerations

The Institutional Review Board of Nam Dinh University of Nursing (347/GCN-HDDD) had approved of the study protocol as well as the questionnaire and written consent form. The participants were informed the study purpose and that they could withdraw from the study at any time during study period. If a participant agreed to involve in the study, a written consent form was obtained. After signing the informed consent form, each participant was given time to read and ask the investigator any questions before filling out the study questionnaire. All personal information of the participants has been anonymously.

## Results

There were 184 clinical nurses, who giving directly care for inpatients in clinical departments, working at NHTD during the study period. Of these, 165 nurses met the inclusion criteria and were enrolled in the study (89.7% of the total study population). The remaining 19 nurses refused to participate in the study. The majority of participants (97.6%) were ≤ 40 years old, 85.5% were female, 60.6% had an associate degree, 75.0% had nursing experience of ≤ 10 years, 81.2% were married, 58.8% cared for children under 5 years old, 68.5% had 7 days or more on duty per month, 53.3% worked in emergency and intensive care departments, 58.8% had cared for COVID-19 patients, 52.7% were satisfied with their job, about one third had an income decrease, and 26.7% were worried about COVID-19 infection (Table 2).

**Table 2:** Socio-demographic and work environment characteristics of participants (n = 165).

Characteristics		Number of patients	
		n	%
Age (in year)	< 30	88	53.3
	30–40	73	442
	> 40	4	2.4
Gender	Male	24	14.5
	Female	141	85.5
Marital status	Married	134	81.2
	Not married, divorced	31	18.8
Having children < 5 years	Yes	97	58.8
	No	68	41.2
Chronic diseases	Yes	24	14.5
	No	141	85.5
Graduate degree	Associate	101	61.2
	Bachelor	64	38,8
Nursing experience time	< = 5 years	62	37.6
	> 5 years and < = 10 years	61	37.0
	> 10 years	42	25.5
Departments	Emergency and intensive care	88	53.3
	The others	77	46.7
Days on duty per month	No	26	15.8
	< 7 days	26	15.8
	> = 7 days	113	68.5
COVID-19 patient care	Yes	97	58.8
	No	68	41.2
Worry about COVID-19 infection	Yes	44	26.7
	Not yet	121	73.3
Feel severe duty for patients	Yes	56	33.9
	Not yet	109	66.1
Job satisfaction	Yes	87	52.7
	Not yet	78	47.3
Lower Income	Yes	58	35.2
	No	107	64.8

### Prevalence of occupational stress

**Fig 1:**
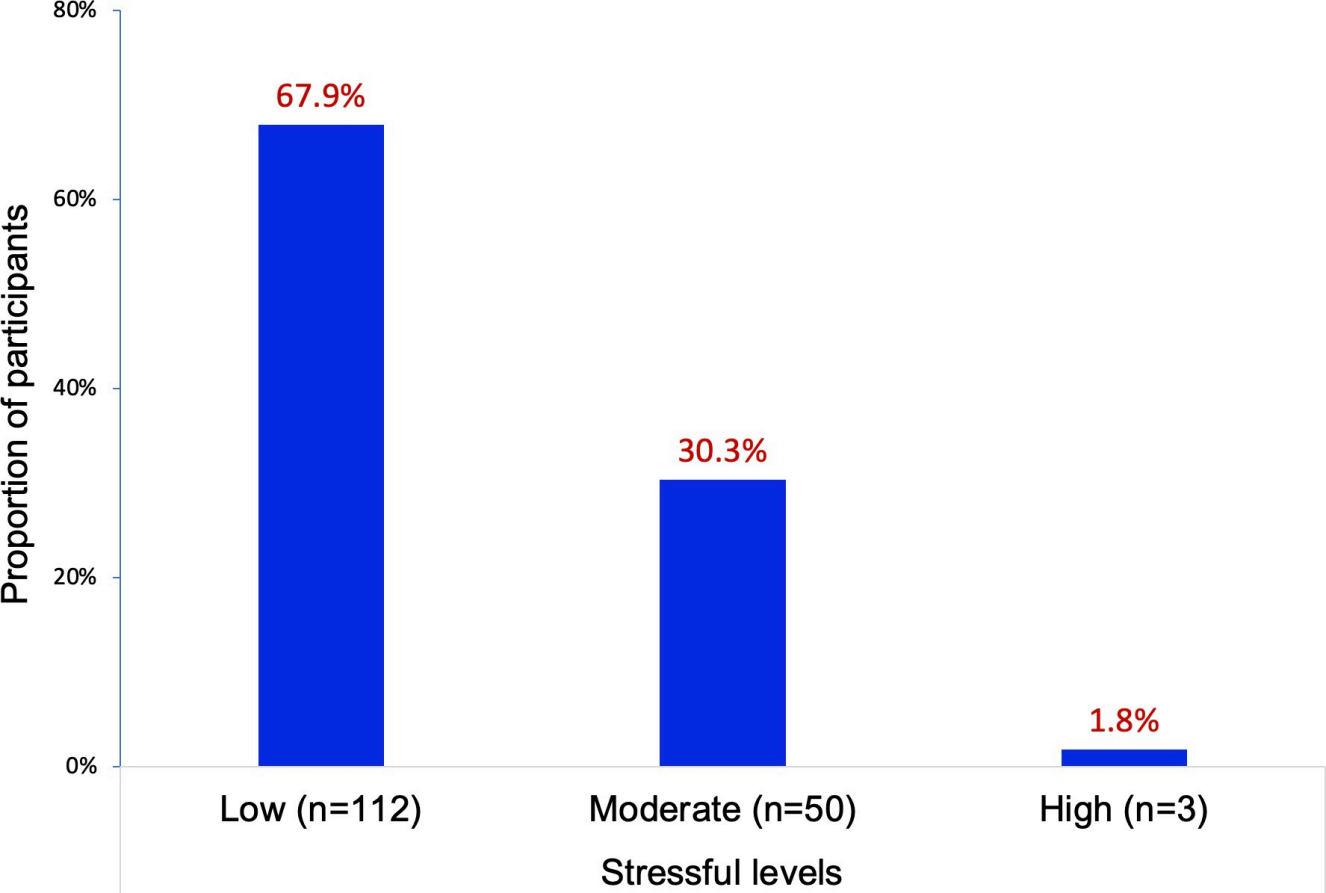
Proportion of stressful levels among participants.

Among 165 participants, there were 112 (67.9%) nurses having low stressful level, 50 (30.3%) nurses having moderate stressful level, and 3 (1.8%) nurses having high stressful level (Fig 1). Occupational stress was perceived in 53 nurses who having moderate or high stressful levels, accounted for 32.1% of participants.

**Fig 2:**
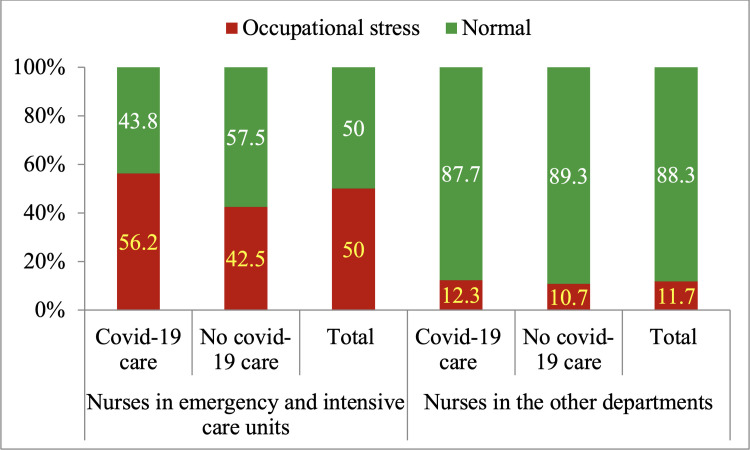
Occupational stress proportion by departments and COVID-19 patient care.

Prevalence of occupational stress among nurses caring for COVID-19 patients (34.0%) was slightly higher than that among nurses who did not care for COVID-19 patients (29.4%). However, this difference was not statistically significant (p = 0.540). In subgroup analysis, the proportion of participants experiencing occupational stress at emergency and intensive care units was 56.3% for those who directly cared for COVID-19 patients and 42.5% for those who did not care for COVID-19 patients. The proportions in the other departments were 12.2% and 10.7%, respectively. These differences between those who directly care for COVID-19 patients and those who did not, were not statistically significant either. The difference in proportion of occupational stress between the emergency and intensive care units (50.0%) and the other departments (11.7%), on the other hand, was statistically significant with p < 0.001 (Fig 2).

**Most stressful factors of ENSS.** The mean ENSS (SD) score for all participants was 1.79 (0.47). This revealed a mild stress level in all participants. The most stressful subscales were patients and families (mean score 1.97 points), followed by Death and dying and Inadequate emotional preparation (both with a mean score of 1.88 points), while the least stressful subscale was Discrimination (mean score of 1.40 points) (Table 3).

**Table 3:** Mean value of ENSS and subscales.

ENSS Subscale	Mean score (SD)
Patients and families	1.97 (0.56)
Death and dying	1.88 (0.51)
Inadequate emotional preparation	1.88 (0.53)
Uncertainty concerning treatment	1.87 (0.56)
Workload	1.82 (0.52)
Conflict with Physicians	1.81 (0.52)
Problems with supervisors	1.62 (0.56)
Problems with peer support	1.59 (0.50)
Discrimination	1.40 (0.63)
**Total ENSS**	**1.79 (0.47)**

**Fig 3:**
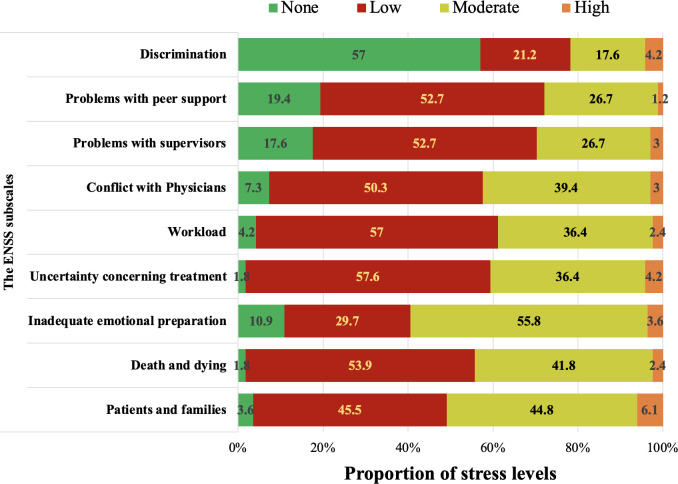
Stressful levels of participants according to the ENSS subscales.

The stressful levels for the ENSS subscales are presented in Fig 3. The proportion of participants with occupational stress (reported moderate or high stressful level) ranged from 21.8% to 59.4%. The most stressful factors were Inadequate emotional preparation (59.4%), followed by Patients and families (50.9%) and Death and dying (44.2%). Discrimination was the least stressful (21.8%).

There were 9 items with a mean score of 2.00 points or higher among the 57 items (designated stress items). Four of these items were in the subscale Patients and families, and two were in the subscale Death and dying. Being exposed to hazards was the most stressful factor, followed by Patients’ unreasonable demands and Helplessness, with no improvement (Table 4).

**Table 4:** The most stressor items in ENSS.

Most stress items	Response frequency of stress level				Mean score (SD)
	Never n (%)	Sometime n (%)	Usually n (%)	Always n (%)	
Being exposed to hazards	22 (13.3)	78 (47.3)	46 (27.9)	19 (11.5)	2.38 (0.86)
Patient’s unreasonable demands	22 (13.3)	95 (57.6)	40 (24.2)	8 (4.8)	2.21 (0.73)
Helpless, no improvement	21 (12.7)	97 (58.8)	42 (25.5)	5 (3.0)	2.19 (0.69)
Unreasonable demands by Patient’s families	32 (19.4)	88 (53.3)	35 (221.2)	10 (6.1)	2.14 (0.80)
Not enough staff in unit	41 (24.8)	74 (44.8)	43 (26.1)	7 (4.2)	2.10 (0.82)
Dealing with violent patients	48 (29.1)	67 (40.6)	39 (23.6)	11 (6.7)	2.08 (0.89)
Dealing with abusive patients	39 (23.6)	85 (51.5)	33 (20.0)	8 (4.8)	2.06 (0.79)
Criticism by a physician	33 (20.0)	100 (60.6)	26 (15.8)	6 (3.6)	2.03 (0.71)
Painful procedures	24 (14.5)	115 (67.9)	24 (14.5)	2 (1.2)	2.02 (0.58)

### Factors associated with nurses’ occupational stress

Six factors (work departments, days on duty per month, worry about COVID-19 infection, feeling heavy duty for patients, job satisfaction, income decrease) were found to be significantly associated with occupational stress in univariate analysis. COVID-19 patient care increased occupational stress; however, the difference was not statistically significant. A multivariate logistic regression analysis revealed that only three risk factors were significantly associated with nurses’ occupational stress. Nurses working in emergency and intensive care units had a higher risk of occupational stress than nurses working in other departments (OR = 4.97, 95% CI (1.82–13.52)). Nurses who felt more heavy duty for patients (usually or more) had a higher risk of occupational stress than those who felt less (OR = 3.17, 95% CI (1.19–8.43)), and nurses with lower income had a higher risk of occupational stress than those who did not (OR = 3.03, 95% CI (1.26–7.27)) (Table 5).

**Table 5:** Factors associated with nurses’ occupational stress.

Risk factors	n (%)	Crude OR (95% CI)	p	Adjusted OR (95% CI)	p
***Age***					
< 30 years old (n = 88)	29 (33.0)	1.08 (0.56–2.09)	0.806	1.20 (0.35–4.10)	0.768
> = 30 years old (n = 77)	24 (31.2)				
***Gender***					
Male (n = 24)	8 (33.3)	1.07 (0.42–2.67)	0.891	0.52 (0.16–1.71)	0.282
Female (n = 141)	45 (31.9)				
***Marital status***					
Married (n = 134)	42 (31.3)	0.83 (0.36–1.89)	0.657	0.80 (0.22–2.88)	0.733
Single or divorced (n = 31)	11 (35.5)				
***Caring for children < 5 years***					
Yes (n = 97)	34 (35.1)	1.39 (0.71–2.73)	0.336	1.18 (0.39–3.60)	0.768
No (n = 68)	19 (27.9)				
***Chronic diseases***					
Yes (n = 24)	4 (33.3)	1.07 (0.42–2.67)	0.891	0.77 (0.25–2.33)	0.640
No (n = 141)	45 (31.9)				
***Educational level***					
Associate (n = 101)	29 (28.7)	0.67 (0.34–1.30)	0.240	0.57 (0.23–1.46)	0.245
Bachelor (n = 64)	24 (37.5)				
***Nursing experience***					
≤ 5 years (n = 62)	19 (30.6)	1.41 (0.58–3.45)	0.447	1.66 (0.32–8.68)	0.548
> 5 years ‐ 10 years (n = 61)	24 (39.3)	2.08 (0.86–4.97)	0.102	1.71 (0.48–6.12)	0.407
> 10 years (n = 42)	10 (23.8)	Reference			
***Work departments***					
Emergency and critical care (n = 88)	44 (50.0)	7.56 (3.36–17.00)	<0.001	4.97 (1.82–13.52)	0.002
The others (n = 77)	9 (11.7)	Reference			
***Days on duty per month***					
None (n = 26)	12 (46.2)	6.57 (1.57–27.43)	0.010	2.47 (0.30–20.09)	0.397
≥ 7 days (n = 113)	38 (33.6)	3.88 (1.10–13.76)	0.035	1.66 (0.26–10.53)	0.591
< 7 days (n = 26)	3 (11.5)	Reference			
***Direct Care for COVID-19 patient***					
Yes (n = 97)	33 (34.0)	1.24 (0.63–2.42)	0.533	1.89 (0.70–5.13)	0.209
No (n = 68)	20 (29.4)	Reference			
***Worry about COVID-19 infection***					
Yes (n = 44)	21 (47.7)	2.54 (1.24–5.20)	0.011	1.02 (0.36–2.83)	0.975
Not yet (n = 121)	32 (26.4)	Reference			
***Having encouragement***					
Sometime or less (n = 55)	22 (40.0)	1.70 (0.86–3.36)	0.127	2.20 (0.86–5.66)	0.101
Usually or more (n = 110)	31(28.2)	Reference			
***Feeling heavy duty for patients***					
Usually or more (n = 56)	27 (48.2)	2.97 (1.50–5.89)	0.002	3.17 (1.19–8.43)	0.021
Sometime or less (n = 109)	26 (23.9)	Reference			
***Job satisfaction***					
Not yet (n = 78)	36 (46.2)	3.53 (1.77–7.05)	< 0.001	2.42 (0.98–5.96)	0.054
Yes (n = 87)	17 (19.5)	Reference			
***Income decrease***					
Yes (n = 58)	33 (56.9)	5.74 (2.82–11.70)	<0.001	3.03 (1.26–7.27)	0.013
No (n = 107)	20 (18.7)	Reference			

## Discussion

This is the first study of occupational stress among all clinical nurses at the National Hospital for Tropical Diseases, a tertiary infectious specialty hospital and the largest designated hospital for COVID-19 patients in North Vietnam.

The overall occupational stress prevalence of clinical nurses at NHTD during the third wave of the COVID-19 epidemic was 32.1%. Prevalence of occupational stress accounted for 50% of the nurses working at emergency and intensive care units. While, this proportion was just 11.7% of nurses in the other departments. The prevalence of occupational stress among nurses caring for COVID-19 patients was somewhat higher than that among nurses who did not. However, the difference was not statistically significant.

The nurses’ occupational stress prevalence found in our study is significantly higher than reports from some other studies. In a 2015 survey of 600 nurses at Viet Duc Hospital, a Vietnamese tertiary hospital, 18.5% reported occupational stress [14]. In a 2019 survey of 347 nurses working in internal departments at 108 Military Hospital, also a Vietnamese tertiary hospital, occupational stress was 19.6% [12]. The differences between findings from these studies and our study is obviously evidence for impact of third wave COVID-19 epidemic on nurses’ occupational stress because these two hospitals are also tertiary hospitals in the North of Vietnam. The difference may be from different instruments used to evaluate stress condition; both these studies used DASS -21 while our study using ENSS. This explanation is supported by report of 17.9% nurses in two tertiary hospitals having stress by DASS-21 during the first wave of COVID-19 in Vietnam [19]. Two other studies also using DASS-21 to evaluate mental health of frontline healthcare workers during the first and second wave of COVID-19 in Vietnam just reported stress rate of 8.0% and 7.7%, respectively participants [20, 23]. This disparity could be attributed to the survey’s use of a different instrument (DASS-21) and its participants, which included physicians, nurses, and other professionals. Furthermore, there were few COVID-19 patients during the first and second wave of COVID-19, and all cases had mild or no symptoms. Another study of 863 healthcare workers in China from February 23^rd^ to March 5^th^ in 2020 using the DASS scale reported 8.6% healthcare workers having stress [24]. This low stress rate may be explained apart by using different instrument (DASS-21) and the participants came from hospitals in seven geographical regions of China. There may be other factors as number and severity of COVID-19 patients but exact information is not mentioned in the report.

However, our finding of nurses’ occupational stress rate was lower than reports from some studies conducted both before and during COVID-19 appearance. A study using DASS-21 instrument conducted in 2017 on 191 clinical nurses at Hanoi Medical University Hospital found that 35.1% of participants experienced occupational stress [13]. The disparity between studies could not be appropriately explained by different instruments, because all studies using DASS-21 above found lower stress prevalence. This could be due to difference in the working environment as Hanoi Medical University Hospital operating like private hospital with higher demand on its staff. One study on 746 healthcare workers in Danang city, in the Middle of Vietnam, reported 44.6% participants having stress [21]. This was an online survey conducted during the second wave of COVID-19 in Danang, the hotspot of COVID-19 in Vietnam at that period. This could be explained by the manner in which COVID-19 occurred there. COVID-19 epidemic was discovered unexpectedly in a department of Danang General Hospital, then spread to other departments and communities. This resulted in an overburdened healthcare system in Danang, which caused chaos because the crisis had not been planned for. Another study of 376 nurses in four governmental hospitals in Harar city, Ethiopia, in March 2015 found that nurses’ occupational stress accounted for 66.2% of participants [1]. This study used the same instrument and definition of occupational stress as in our study. The different findings may be due to stress perception varied with changing time and differences in cultural, professional, and working environment.

Occupational stress can have a severe impact on nurses’ quality of life, resulting in poor patient care [2]. As a result, controlling nurses’ occupational stress is critical for maintaining nurses’ quality of life and mental health while also improving patient care quality. Particularly during pandemics such as COVID-19, when there is an immediate demand for a large number of nurses [25].

The most stressful sources of occupational stress for nurses were Inadequate emotional preparation, Patients and families, and Death and Dying (Table 3). A survey of nurses’ occupational stress in Vietnam conducted by ENSS found that Death and Dying, and Patients and families, but not Inadequate emotional preparation were the most stressful factors for nurses [16, 17]. This could be because a survey was conducted before the occurrence of COVID-19, but our study was conducted during the development stage of the COVID-19 pandemic, when emotional preparation was critical in dealing with COVID-19 patients. The most stressful items in this study, according to item analysis, were being exposed to hazards, the patient’s unreasonable demands, and helplessness with no improvement (Table 4). This could be attributed to the quick exploration of the COVID-19 pandemic, with nurses being the most vulnerable subjects. As a result, these subscales and items should be prioritized in order to reduce occupational stress among nurses.

The COVID-19 pandemic caused many problems for healthcare workers and the healthcare system, including occupational stress [25–27]. This study found that nurses who had personally cared for COVID-19 patients had a slightly higher rate of occupational stress than nurses who had not. However, the difference was not statistically significant. This finding differed from other studies [20, 21], although it was consistent with the findings of a survey conducted in Qatar in late 2020 [28]. This could be explained by the fact that the NHTD staff had already been trained in the knowledge and abilities required to manage COVID-19 patients and COVID-19 pandemic. Furthermore, because the study was conducted during the third wave of COVID-19 in Vietnam, the nurses had prior experience caring for COVID-19 patients during earlier outbreaks of COVID-19. Otherwise, NHTD is a specialty hospital for infectious diseases, so nurses may have gained good knowledge and abilities from dealing with highly contagious diseases in the past, such as the SARS pandemic in 2003, avian influenza in 2005, and the H1N1 2009 flu. As a result, they may feel more confident in caring for COVID-19 patients. Therefore, COVID-19 has caused little concern among nurses at NHTD. This could also explain why previous studies found that concern about COVID-19 infection was associated with occupational stress [9, 10], but not in this study.

Working in an emergency and intensive care unit was revealed to be one of three independent factors related to a higher rate of nurses’ occupational stress in multivariate logistic regression (OR = 4.97, 95% CI (1.82–13.52)) (Table 5). This finding investigates the fact that high workloads as well as the severity of patients in emergency and intensive care units are the most stressful factors for nurses. This is consistent with the findings of a survey conducted in Harar Eastern, Ethiopia, which found that nurses in intensive care units had the highest risk of occupational stress, with an adjusted OR = 4.5 (1.2–17.7) when compared to nurses in outpatient clinics [1]. The current study’s findings also lend support to findings from a 2020 survey of healthcare workers in 70 French hospitals’ intensive care units, which revealed that intensive care units with higher-intensity had higher levels of overall perceived stress [26]. Another study found that nurses in emergency and intensive care units were vulnerable to psycho-socio-emotional needs [29]. Another risk factor was the personal qualities of nurses. Those who had felt higher heavy duty for patients (usually or more frequently) had a higher risk of occupational stress (OR = 3.17, 95% CI (1.19–8.43)) than those who felt less heavy duty. When establishing a nursing department, this element should be considered. The final risk factor was income decline (OR = 3.03, 95% CI (1.26–7.27)), which was corroborated by the results of a 2020 survey in Turkey [30]. Income reductions had a negative impact on everyone, not just nurses. This factor, however, would be particularly serious for clinical nurses because it could have a negative impact on patient care quality. As a result, it must be concerned with the actual situation.

### Limitations

Our study has several limitations. Firstly, because this was a cross-sectional survey, the direction of causality in associations between risk factors and nurses’ occupational stress is difficult to determine. Secondly, our study only included clinical nurses during the third wave of COVID-19 pandemic in Vietnam. Therefore, the results may not be representative of nurses in other roles, such as those who do not directly care for patients. Finally, because the study was conducted at the National Hospital for Tropical Diseases, a tertiary infection specialty hospital, the staff and nurses had more experience and perspectives to deal with many communicable diseases and epidemics, such as the severe acute respiratory syndrome in early 2003, the outbreak of H5N1 avian flu in late 2003, the pandemic of H1N1 flu in 2009, and the measle outbreak in early 2014. Therefore, the influence of CCOVID-19 on nurses’ occupational stress in this study may be underestimated for nurses in other Vietnamese healthcare facilities.

## Conclusion

The overall occupational stress level of clinical nurses at NHTD was at a mild level. Nearly one-third of nurses experienced moderate and severe stress levels. Occupational stress was reported by 50% of clinical nurses in the emergency and intensive care units, compared to 11.7% of nurses in other departments. The most stressors were associated with the subscales “Inadequate emotional preparation”, “patients and families” and “death and dying”. Working in an emergency and intensive care unit, feeling heavy duty for patients on a regular or frequent basis, and a drop in salary were the most common factors related to a higher risk of occupational stress among nurses. In this study, COVID-19 patient care resulted in a slight increase in the proportion of nurses experiencing occupational stress, but this increase was not statistically significant. However, given the context of this study, this should be approached with caution.

## Supporting information

S1 File Vietnamese-language questionnaire. (DOCX)

S2 File English-language questionnaire. (DOCX)

S1 Data (XLSX)
