# Supplementary material for: Occupational stress and associated factors among clinical nurses caring for COVID-19 patients in a Vietnamese tertiary hospital
Source: PLoS One. 2024 Aug 15;19(8):e0309028. doi: 10.1371/journal.pone.0309028 (PMC11326648; doi:10.1371/journal.pone.0309028)
Supplement: S1 File — (DOCX) [file pone.0309028.s001.docx]

**PHIẾU KHẢO SÁT CĂNG THẲNG NGHỀ NGHIỆP VÀ MỘT SỐ YẾU TỐ LIÊN QUAN Ở ĐIỀU DƯỠNG LÂM SÀNG BỆNH VIỆN BỆNH NHIỆT ĐỚI TRUNG ƯƠNG TRONG ĐỢT DỊCH BỆNH COVID-19 THỨ BA**

Mã số: ___________ Ngày khảo sát: …../ …. /2021

**I. THÔNG TIN CÁ NHÂN**

*Anh/chị vui lòng đánh dấu “x” vào ô trống hoặc điền thông tin vào chỗ “…”*

1. Giới tính:

| 1.Nam | 🖵 |
| --- | --- |
| 2.Nữ | 🖵 |

2. Tuổi: ………(năm)

3. Trình độ chuyên môn cao nhất hiện tại

| 1.Trung cấp | 🖵 |
| --- | --- |
| 2.Cao đẳng | 🖵 |
| 3.Đại học | 🖵 |
| 4.Sau đại học | 🖵 |

4. Thâm niên công tác (tổng thời gian làm việc tại các bệnh viện) ....... năm.

5. Tình trạng hôn nhân:

| 1.Chưa kết hôn | 🖵 |
| --- | --- |
| 2.Đã kết hôn | 🖵 |
| 3.Ly hôn/ly thân/Độc thân | 🖵 |

6. Anh/chị làm việc tại khoa:

| 1.Khoa Cấp cứu, Hồi sức tích cực | 🖵 |
| --- | --- |
| 2.Khoa khác | 🖵 |

7. Anh/chị có bị bệnh mãn tính

| 1.Có | 🖵 |
| --- | --- |
| 2.Không | 🖵 |

8. Anh/chị có đang nuôi con nhỏ dưới 5 tuổi

| 1.Có | 🖵 |
| --- | --- |
| 2.Không | 🖵 |

9. Số buổi trực trong một tháng của Anh/chị là.....buổi

**II. CĂNG THẲNG TRONG CÔNG VIỆC THEO THANG ĐIỂM ENSS**

*Những nội dung dưới đây là những tình huống trong công việc hàng ngày của điều dưỡng. Anh, chị đưa ra cảm nhận của mình trong các tình huống đó. Mỗi câu anh chị đọc và khoanh tròn tương ứng theo mức độ cảm nhận của bản thân.* *Trường hợp có yếu tố “không áp dụng” với bản thân thì Anh/chị để trống không lựa chọn*.

**Các mức độ:**

1. Chưa bao giờ căng thẳng

2. Thỉnh thoảng căng thẳng

3.Thường xuyên căng thẳng

4. Luôn luôn căng thẳng

| **TT** | **Nội dung** | **Mức độ** | | | |
| --- | --- | --- | --- | --- | --- |
| 1 | Thực hiện các quy trình mà người bệnh cảm thấy đau đớn | 1 | 2 | 3 | 4 |
| 2 | Bị chỉ trích bởi bác sĩ | 1 | 2 | 3 | 4 |
| 3 | Cảm giác không được chuẩn bị đầy đủ để giúp đỡ nhu cầu tình cảm cho gia đình người bệnh | 1 | 2 | 3 | 4 |
| 4 | Thiếu cơ hội nói chuyện cởi mở với các vấn đề liên quan khác vói đồng nghiệp các khoa về các vấn đề liên quan đến nơi làm việc | 1 | 2 | 3 | 4 |
| 5 | Xung đột với người giám sát | 1 | 2 | 3 | 4 |
| 6 | Thông tin không đầy đủ từ một bác sĩ về tình trạng y tế của người bệnh | 1 | 2 | 3 | 4 |
| 7 | Người bệnh đưa ra những yêu cầu vô lý | 1 | 2 | 3 | 4 |
| 8 | Bị quấy rối về tình dục | 1 | 2 | 3 | 4 |
| 9 | Cảm thấy bất lực trong trường hợp người bệnh không cải thiện | 1 | 2 | 3 | 4 |
| 10 | Xung đột với bác sĩ | 1 | 2 | 3 | 4 |
| 11 | Bị người bệnh hỏi một câu hỏi mà tôi không có câu trả lời thỏa đáng | 1 | 2 | 3 | 4 |
| 12 | Thiếu cơ hội chia sẻ kinh nghiệm và cảm xúc với các nhân viên khác trong môi trường làm việc | 1 | 2 | 3 | 4 |
| 13 | Không thể đoán trước được nhân lực và kế hoạch làm việc | 1 | 2 | 3 | 4 |
| 14 | Một bác sĩ ra chỉ định điều trị có vẻ không phù hợp cho người bệnh | 1 | 2 | 3 | 4 |
| 15 | Gia đình người bệnh đưa ra những yêu cầu vô lý | 1 | 2 | 3 | 4 |
| 16 | Bị phân biệt đối xử về chủng tộc hoặc dân tộc | 1 | 2 | 3 | 4 |
| 17 | Lắng nghe bệnh nhân kể về cái chết cận kề của họ | 1 | 2 | 3 | 4 |
| 18 | Sợ mắc sai lầm khi điều trị người bệnh | 1 | 2 | 3 | 4 |
| 19 | Cảm thấy không thỏa đáng chuẩn bị sẵn sàng để giúp đỡ các nhu cầu tình cảm của người bệnh | 1 | 2 | 3 | 4 |
| 20 | Thiếu cơ hội để bày tỏ với người khác trong đơn vị về cảm xúc tiêu cực của bản thân với người bệnh | 1 | 2 | 3 | 4 |
| 21 | Khó làm việc với một điều dưỡng hoặc nhiều điều dưỡng khi thực hiện công việc cấp cứu | 1 | 2 | 3 | 4 |
| 22 | Khó làm việc với một điều dưỡng hoặc nhiều điều dưỡng khi không thực hiện công việc cấp cứu | 1 | 2 | 3 | 4 |
| 23 | Không đủ thời gian để hỗ trợ tinh thần cho người bệnh | 1 | 2 | 3 | 4 |
| 24 | Một bác sĩ không có mặt trong tình huống cấp cứu | 1 | 2 | 3 | 4 |
| 25 | Bị đổ lỗi cho bất cứ điều gì sai sót | 1 | 2 | 3 | 4 |
| 26 | Bị phân biệt đối xử liên quan đến giới tính | 1 | 2 | 3 | 4 |
| 27 | Chứng kiến cái chết của người bệnh | 1 | 2 | 3 | 4 |
| 28 | Bất đồng liên quan đến việc điều trị cho người bệnh | 1 | 2 | 3 | 4 |
| 29 | Cảm thấy không được đào tạo đầy đủ cho những việc phải làm | 1 | 2 | 3 | 4 |
| 30 | Thiếu hỗ trợ của người giám sát trực tiếp | 1 | 2 | 3 | 4 |
| 31 | Bị chỉ trích bởi người giám sát | 1 | 2 | 3 | 4 |
| 32 | Không đủ thời gian để hoàn thành tất cả các nhiệm vụ điều dưỡng của mình | 1 | 2 | 3 | 4 |
| 33 | Không biết người bệnh và người nhà người bệnh cần được thông báo những gì về tình trạng bệnh và cách điều trị | 1 | 2 | 3 | 4 |
| 34 | Là người phải đối phó với người nhà người bệnh | 1 | 2 | 3 | 4 |
| 35 | Phải đối phó với người bệnh bạo lực | 1 | 2 | 3 | 4 |
| 36 | Tiếp xúc với những nguy hiểm cho sức khỏe và an toàn bản thân | 1 | 2 | 3 | 4 |
| 37 | Chứng kiến cái chết của người bệnh mà bạn đã phát triển mối quan hệ thân thiết | 1 | 2 | 3 | 4 |
| 38 | Đưa ra quyết định liên quan đến người bệnh khi bác sĩ không có mặt | 1 | 2 | 3 | 4 |
| 39 | Người phụ trách không đủ kinh nghiệm | 1 | 2 | 3 | 4 |
| 40 | Thiếu hỗ trợ của người quản lý điều dưỡng | 1 | 2 | 3 | 4 |
| 41 | Yêu cầu quá nhiều công việc không liên quan đến điều dưỡng, chẳng hạn như công việc văn thư | 1 | 2 | 3 | 4 |
| 42 | Không đủ nhân lực để bao quát công việc của đơn vị | 1 | 2 | 3 | 4 |
| 43 | Không nắm chắc về hoạt động cũng như chức năng của các thiết bị chuyên dụng | 1 | 2 | 3 | 4 |
| 44 | Phải đối phó với người bệnh có hành vi lạm dụng | 1 | 2 | 3 | 4 |
| 45 | Không có đủ thời gian để đáp ứng nhu cầu của người nhà người bệnh | 1 | 2 | 3 | 4 |
| 46 | Chịu trách nhiệm về những việc mà tôi không kiểm soát được | 1 | 2 | 3 | 4 |
| 47 | Bác sĩ không có mặt khi người bệnh tử vong | 1 | 2 | 3 | 4 |
| 48 | Phải tổ chức công việc của bác sĩ | 1 | 2 | 3 | 4 |
| 49 | Thiếu hỗ trợ của các cấp lãnh đạo khác | 1 | 2 | 3 | 4 |
| 50 | Khó làm việc với điều dưỡng khác giới | 1 | 2 | 3 | 4 |
| 51 | Yêu cầu của việc phân loại người bệnh | 1 | 2 | 3 | 4 |
| 52 | Phải đối mặt với sự ngược đãi từ gia đình người bệnh | 1 | 2 | 3 | 4 |
| 53 | Chứng kiến người bệnh đau khổ | 1 | 2 | 3 | 4 |
| 54 | Bị chỉ trích từ điều dưỡng trưởng | 1 | 2 | 3 | 4 |
| 55 | Phải làm việc trong thời gian nghỉ | 1 | 2 | 3 | 4 |
| 56 | Không biết liệu gia đình người bệnh có tố cáo bạn về việc chăm sóc không đầy đủ | 1 | 2 | 3 | 4 |
| 57 | Phải đưa ra quyết định dưới áp lực | 1 | 2 | 3 | 4 |

**III. CÁC YẾU TỐ LIÊN QUAN ĐẾN MÔI TRƯƠNG LÀM VIỆC VÀ THU NHẬP**

*Bản thân là điều dưỡng Bệnh viện Bệnh Nhiệt đới Trung Ương, nơi tuyến đầu chống dịch, anh/chị vui lòng cho biết cảm nhận về các điều kiện và môi trường làm việc. Anh/chị hãy trả lời câu hỏi bằng cách tích “X” vào đáp án phù hợp với mình.*

**Yếu tố tinh thần trong môi trường làm việc:**

Câu 1. Anh/ chị đã bao giờ lo lắng về nguy cơ phơi nhiễm với virut SARS-CoV 2 trong quá trình làm việc tại bệnh viện?

| 1.Chưa bao giờ | 🖵 |
| --- | --- |
| 2.Thỉnh thoảng | 🖵 |
| 3.Thường xuyên | 🖵 |
| 4.Luôn luôn | 🖵 |

Câu 2. Trong giai đoạn bệnh viện chống dịch COVID- 19, Anh/chị có nhận được sự chia sẻ về tinh thần ra sao?

| 1. Chưa bao giờ | 🖵 |
| --- | --- |
| 2.Thỉnh thoảng | 🖵 |
| 3. Thường xuyên | 🖵 |
| 4. Luôn luôn | 🖵 |

Câu 3. Anh/chị thấy nhận thấy trách nhiệm của mình với người bệnh nặng nề không?

| 1. Chưa bao giờ | 🖵 |
| --- | --- |
| 2.Thỉnh thoảng | 🖵 |
| 3. Thường xuyên | 🖵 |
| 4. Luôn luôn | 🖵 |

Câu 4. Anh/chị có thấy hài lòng với công việc hiện tại không?

| 1. Chưa bao giờ | 🖵 |
| --- | --- |
| 2.Tương đối hài lòng | 🖵 |
| 3. Hài lòng | 🖵 |
| 4. Rất hài lòng | 🖵 |

Câu 5. Anh/chị đã từng chăm sóc người bệnh COVID- 19 chưa?

| 1. Có | 🖵 |
| --- | --- |
| 2. Không | 🖵 |

Câu 6. Thu nhập của Anh/chị trong giai đoạn bùng phát dịch COVID-19 đến hiện tại có bị ảnh hưởng không?

| 1. Duy trì ổn định hoặc ít thay đổi | 🖵 |
| --- | --- |
| 2. Giảm đi đáng kể | 🖵 |
