# Supplementary material for: Occupational stress and associated factors among clinical nurses caring for COVID-19 patients in a Vietnamese tertiary hospital
Source: PLoS One. 2024 Aug 15;19(8):e0309028. doi: 10.1371/journal.pone.0309028 (PMC11326648; doi:10.1371/journal.pone.0309028)
Supplement: S2 File — (DOCX) [file pone.0309028.s002.docx]

**QUESTIONNAIRE**

**OCCUPATION STRES AND ASSOCIATED FACTOR AMONG CLINICAL NURSES IN NATIONAL HOSPITAL FOR TROPICAL DISEASES DURING THE THIRD WAVE OF COVID-19**

Form code: ___________ survey date: ….. / …../2021

**I. GENERAL INFORMATION**

*Please fill out the information in space or cross (x) to answer the following questions*

1. Gender:

| 1.Male | 🖵 |
| --- | --- |
| 2.Female | 🖵 |

2. Age: ………(year)

3. What is your current level of education

| 1. Intermediate | 🖵 |
| --- | --- |
| 2. Associate | 🖵 |
| 3. Bachelor | 🖵 |
| 4. Post graduate | 🖵 |

4. How many years of nursing work have you have until now? ....... .. years.

5. Marital status:

| 1. Single | 🖵 |
| --- | --- |
| 2. Marriage | 🖵 |
| 3. Divorce/widow | 🖵 |

6. Department:

| 1. Emergency or intensive care units | 🖵 |
| --- | --- |
| 2. The other departments | 🖵 |

7. Do you have chronic diseases

| 1. Yes | 🖵 |
| --- | --- |
| 2. No | 🖵 |

8. Are you caring for children <= 5 years old

| 1. Yes | 🖵 |
| --- | --- |
| 2. No | 🖵 |

9. Number of days on duty per month: ......... days

**II. OCCUPATIONAL STRESS BY THE EXTENDED NURSING STRESS SCALE**

Please read each statement and circle a number 1, 2, 3 or 4 which indicates how much the statement applied to you in daily work according to your perception. There are no right or wrong answers. Do not spend too much time on any statement. In case the statement is not apply to you, please not circle any number.

**Stress levels:**

1. Never stressful

2. Sometime stressful

3. Usually stressful

4. Always stressful

| **ON** | **Items** | **Stressful levels** | | | |
| --- | --- | --- | --- | --- | --- |
| 1 | Painful procedures | 1 | 2 | 3 | 4 |
| 2 | Criticism by a physician | 1 | 2 | 3 | 4 |
| 3 | Feeling unprepared to help family with emotional needs | 1 | 2 | 3 | 4 |
| 4 | Lack of opportunity to talk with other personel | 1 | 2 | 3 | 4 |
| 5 | Conflict with a supervisor | 1 | 2 | 3 | 4 |
| 6 | Uncertainty about treatment | 1 | 2 | 3 | 4 |
| 7 | Patient’s unreasonable demands | 1 | 2 | 3 | 4 |
| 8 | Being sexually harassed | 1 | 2 | 3 | 4 |
| 9 | Helpless, no improvement | 1 | 2 | 3 | 4 |
| 10 | Conflict with a physician | 1 | 2 | 3 | 4 |
| 11 | Having no answer for patient | 1 | 2 | 3 | 4 |
| 12 | Lack of opportunity to share experience with personnel | 1 | 2 | 3 | 4 |
| 13 | Unpredictable staffing/schedule | 1 | 2 | 3 | 4 |
| 14 | Physician ordering inappropriate | 1 | 2 | 3 | 4 |
| 15 | unreasonable demands by Patient’s families | 1 | 2 | 3 | 4 |
| 16 | Discrimination against race | 1 | 2 | 3 | 4 |
| 17 | Talking to patient about death | 1 | 2 | 3 | 4 |
| 18 | Fear of making a mistake | 1 | 2 | 3 | 4 |
| 19 | Unprepared to help patient with emotional needs | 1 | 2 | 3 | 4 |
| 20 | Lack of oppotunity to express negative feelings about patients | 1 | 2 | 3 | 4 |
| 21 | Difficulty with another nurse in immediate work seting | 1 | 2 | 3 | 4 |
| 22 | Difficulty with another nurse outside immediate work seting | 1 | 2 | 3 | 4 |
| 23 | No time to give patient support | 1 | 2 | 3 | 4 |
| 24 | Physician not present in a medical emergency | 1 | 2 | 3 | 4 |
| 25 | Being blamed for things that go wrong | 1 | 2 | 3 | 4 |
| 26 | Discrimination against sex | 1 | 2 | 3 | 4 |
| 27 | Death of a patient | 1 | 2 | 3 | 4 |
| 28 | Disagreement about treatment | 1 | 2 | 3 | 4 |
| 29 | Feeling inadequately trained | 1 | 2 | 3 | 4 |
| 30 | Lack of support from immediate supervisor | 1 | 2 | 3 | 4 |
| 31 | Criticism by a suppervisor | 1 | 2 | 3 | 4 |
| 32 | Insufficient time to finish tasks | 1 | 2 | 3 | 4 |
| 33 | Not knowing what patient or family should be told | 1 | 2 | 3 | 4 |
| 34 | Having to handle patient’s families | 1 | 2 | 3 | 4 |
| 35 | Dealing with violent patients | 1 | 2 | 3 | 4 |
| 36 | Being exposed to hazards | 1 | 2 | 3 | 4 |
| 37 | Death of a close patient | 1 | 2 | 3 | 4 |
| 38 | Making a decision without physician | 1 | 2 | 3 | 4 |
| 39 | Being in charge with inadequate experience | 1 | 2 | 3 | 4 |
| 40 | Lack of support from nursing administration | 1 | 2 | 3 | 4 |
| 41 | Too many non-nursing tasks | 1 | 2 | 3 | 4 |
| 42 | Not enough staff in unit | 1 | 2 | 3 | 4 |
| 43 | Uncertainty about operation and functioning of equipment | 1 | 2 | 3 | 4 |
| 44 | Dealing with abusive patients | 1 | 2 | 3 | 4 |
| 45 | Not enough time to respond to needs of patient’s families | 1 | 2 | 3 | 4 |
| 46 | Accountable for things beyond control | 1 | 2 | 3 | 4 |
| 47 | Physician not present when patient dies | 1 | 2 | 3 | 4 |
| 48 | Organizing doctor’work | 1 | 2 | 3 | 4 |
| 49 | Lack of support from other health care administrators | 1 | 2 | 3 | 4 |
| 50 | Difficulty working with nurses of opposite sex | 1 | 2 | 3 | 4 |
| 51 | Demands of classification system | 1 | 2 | 3 | 4 |
| 52 | Dealing with abuse from patient’s families | 1 | 2 | 3 | 4 |
| 53 | Watching patient suffer | 1 | 2 | 3 | 4 |
| 54 | Criticism by nursing administrator | 1 | 2 | 3 | 4 |
| 55 | Having to work through breaks | 1 | 2 | 3 | 4 |
| 56 | Uncertainty about being reported by patient’s families | 1 | 2 | 3 | 4 |
| 57 | Making decisions under pressure | 1 | 2 | 3 | 4 |

**III. WORKING INVIRONMENT AND INCOME**

*You are clinical nurse in the National Hospital for Tropical Disease, the frontline tertiary hospital for COVID-19 patients and COVID-19 pandemic, please let us know about your feel of working environment and facility. Please cross (x) to an answer that is relevant to your situation.*

1. Have you ever worried about COVID-19 infection during your work?

| 1. Never | 🖵 |
| --- | --- |
| 2. Sometime | 🖵 |
| 3. Usually | 🖵 |
| 4. Always | 🖵 |

2. In wave of COVID-19 period, have you received mental health support?

| 1. Never | 🖵 |
| --- | --- |
| 2. Sometime | 🖵 |
| 3. Usually | 🖵 |
| 4. Always | 🖵 |

3. How often do you feel heavy about your duty for patients?

| 1. Never | 🖵 |
| --- | --- |
| 2. Sometime | 🖵 |
| 3. Usually | 🖵 |
| 4. Always | 🖵 |

4. Do you satisfy with your current job?

| 1. Never | 🖵 |
| --- | --- |
| 2. Fair satisfaction | 🖵 |
| 3. Satisfaction | 🖵 |
| 4. Very satisfaction | 🖵 |

5. Have you already cared for COVID-19 patients?

| 1. Yes | 🖵 |
| --- | --- |
| 2. No | 🖵 |

6. How about your income during the wave of COVID-19?

| 1. Unchange or Increase | 🖵 |
| --- | --- |
| 2. Decrease | 🖵 |
